# Claudin proteins and hemorrhage severity in aneurysmal subarachnoid hemorrhage: Correlation with modified Fisher score but not functional outcome

**DOI:** 10.1007/s10143-025-03829-y

**Published:** 2025-10-20

**Authors:** Gabor Lenzser, Gábor J. Szebeni, Fanni Balogh, Nikolett Gémes, Attila Schwarcz, Peter Csecsei

**Affiliations:** 1 Department of Neurosurgery, Medical School, University of Pecs, 7622, Ret str.2, Pecs, 7624 Hungary; 2 Laboratory of Flow Cytometry, Core Facility, HUN-REN Biological Research Centre, Szeged, Hungary; 3 Department of Internal Medicine, Hematology Centre, Faculty of Medicine, University of Szeged, Szeged, Hungary; 4 Department of Rheumatology and Immunology, Albert Szent-Gyorgyi Medical School and Health Center, University of Szeged, Szeged, Hungary

**Keywords:** Aneurysmal subarachnoid hemorrhage, Claudin-3, Claudin-5, Modified Fisher score

## Abstract

Background: Aneurysmal subarachnoid hemorrhage (aSAH) is a critical condition characterized by blood-brain barrier (BBB) disruption. Tight junction proteins, claudin-3 (CLDN3) and claudin-5 (CLDN5), are key regulators of BBB integrity and may serve as biomarkers of hemorrhage severity. Methods: In this prospective cohort study, 200 patients with aSAH were evaluated. Serum CLDN3 and CLDN5 levels were measured on days 1, 5, and 9 post-ictus and compared with healthy and aneurysm-bearing controls. Patients were stratified by modified Fisher Score (mFS), World Federation of Neurosurgical Societies (WFNS) score, and 3-month modified Rankin Scale (mRS). Associations with complications, including delayed cerebral ischemia (DCI) and infection, were also assessed. Results: CLDN3 and CLDN5 levels were significantly elevated in aSAH patients compared to controls (*p *<0.01 and *p *<0.0001, respectively), with CLDN5 levels consistently higher than CLDN3 at all time points (*p *<0.0001). CLDN5 levels were consistently higher than CLDN3 at all measured time points, and neither CLDN3 nor CLDN5 showed significant temporal variation between days 1, 5, and 9. Both markers showed significant positive correlation with mFS, with higher levels observed in mFS3 and mFS4 groups (*p *<0.05 to *p *<0.0001). No association was found between CLDN3 and 5 levels and WFNS score, 3-month mRS, age, sex, comorbidities, inflammatory markers, or DCI. Conclusion: Elevated serum CLDN3 and especially CLDN5 levels reflect hemorrhage extent and potential BBB disruption in aSAH. Their strong correlation with mFS suggests utility in grading imageological hemorrhagic severity, though not in predicting long-term functional outcomes. CLDN5 emerges as a promising biomarker for early assessment of BBB integrity in neurovascular injury.

**Supplementary Information:**

The online version contains supplementary material available at 10.1007/s10143-025-03829-y.

## Introduction

Aneurysmal subarachnoid hemorrhage (aSAH) is a severe cerebrovascular event characterized by bleeding into the subarachnoid space due to a ruptured intracranial aneurysm. Globally, the incidence of aSAH has declined over recent decades, with current estimates ranging from 6.1 to 9.0 per 100,000 person-years [1]. Notably, higher incidence rates are observed in countries like Japan and Finland [1], and the condition predominantly affects individuals aged 40 to 60 years, with a slightly higher prevalence in females [2].

Despite advancements in medical care, aSAH continues to carry a high mortality rate. Recent data indicate a 30-day mortality rate of approximately 20.4%, with some studies reporting short-term mortality rates around 18.4% and 5-year mortality rates up to 29% [3].

The impact of aSAH extends beyond mortality, as many survivors experience significant long-term neurological deficits. Cognitive impairments, including memory loss and executive dysfunction, are common and can severely affect quality of life [4]. Additionally, only a minority of survivors regain their pre-hemorrhage level of daily functioning [5].​

The Modified Fisher Score (mFS) is a clinical grading system used to assess the severity of aSAH based on the amount and distribution of blood seen on initial CT imaging. The score is based on the degree of blood in the subarachnoid space, ranging from 0 (no blood) to 4 (extensive blood) [6]. Studies support the use of the mFS as a reliable tool for quantifying the extent of hemorrhage in aSAH and for predicting associated neurological complications [7– 8].

The extent or amount of blood in subarachnoid hemorrhage (SAH) is strongly associated with the degree of blood-brain barrier (BBB) injury [9]. Upon aneurysmal rupture, extravasated blood rapidly increases intracranial pressure and extravasates into brain compartments, which disrupts cerebral perfusion and triggers early global ischemia. Hemoglobin and its breakdown products stimulate inflammatory pathways and oxidative cascades that compromise the BBB as early as 30 min post-ictus [10], leading to structural breakdown through tight junction degradation and extracellular matrix damage [11].

BBB maintains homeostatic and autoregulatory functions in the brain, regulating molecular transfer and preventing toxic insults [12]. Structurally, the BBB is formed by endothelial cells, which are supported by astrocytes and pericytes that perform maintenance and repair functions [13]. Claudin-5 (CLDN5) is an endothelial cell-specific member of the claudin family that plays a crucial role in the proper organization of tight junctions and in preserving the integrity of brain microvascular endothelial cells [14]. It is widely recognized for its contribution to the “sealing” function of tight junctions, thereby regulating the permeability of the BBB [15]. CLDN5 seals the intercellular space by forming interactions between its extracellular loops and those of CLDN5 molecules on neighboring endothelial cells. Recent studies suggest that CLDN5 may play a role in various diseases, including Alzheimer’s, edema, hypoglycemia, inflammation, trauma, and tumors [14, 16–18]. Claudin-3 (CLDN3) is a tight junction protein that contributes to the integrity of epithelial and endothelial barriers, including the blood–cerebrospinal fluid barrier, where it regulates paracellular permeability and barrier function​ [19].

Although claudin-5 is the dominant claudin isoform at the BBB, claudin-3 provides additional sealing properties and helps maintain junctional architecture [20]. Brain endothelial cells predominantly express CLDN3 and CLDN5 [21]– [22] and a substantial body of evidence clearly demonstrates the essential role of CLDN3 and CLDN5 in the formation and maintenance of tight junctions at the blood–brain barrier [23]. However, CLDN5 can interact with CLDN3 [24–25]. CLDN3 plays a crucial role in maintaining blood–cerebrospinal fluid barrier (BCSFB) integrity, and its loss at the choroid plexus contributes to increased leukocyte infiltration and exacerbated neuroinflammation in multiple sclerosis [26].

Both CLDN3 and CLDN5 levels can be measured in blood, and their presence is associated with various pathological conditions [27–29]. Moreover, claudin-5 clearly reflects the extent of blood–brain barrier (BBB) damage in various neurological conditions [19]. Our study aims to explore the relationship between clinical and radiological severity scoring systems of subarachnoid hemorrhage and the serum levels of CLDN3 and CLDN5—proteins released during blood-brain barrier (BBB) disruption. By doing so, we seek to clarify the potential correlation between the extent of the hemorrhage and the degree of BBB damage in aneurysmal subarachnoid hemorrhage.

## Materials & methods

### Study design and participants

This was a prospective, multicenter cohort study involving consecutive patients with aneurysmal subarachnoid hemorrhage (aSAH) admitted to the Department of Neurosurgery at the University of Pécs, Hungary, between December 2021 and November 2024. The study was approved by the institutional ethics committee (IV/8468‑1/2021/EKU), and written informed consent was obtained from all participants or their legal representatives. Patients were eligible if they met al.l of the following inclusion criteria: (i) age ≥ 18 years, (ii) diagnosis of spontaneous aneurysmal SAH confirmed by non‑contrast head CT performed within 24 h of ictus, (iii) presence of a ruptured intracranial aneurysm confirmed by CTA or DSA, (iv) underwent aneurysm treatment (endovascular coiling or stent-assisted coiling) within 24 h of admission, (v) complete clinical and radiological data available, including modified Fisher grading on admission. Patients were excluded if they met any of the following exclusion criteria: (i) traumatic SAH or SAH secondary to arteriovenous malformation, (ii) pregnancy, (iii) admission later than 24 h after ictus, (iv) no aneurysm treatment performed, (v) lack of written informed consent, (vi) known systemic diseases that could influence biomarker levels, including malignant tumors, chronic liver or renal insufficiency, chronic lung disease, chronic neurological disorders, inflammatory bowel disease, or chronic gastrointestinal disorders and (vii) clinical or laboratory evidence of acute or chronic infection (including SARS‑CoV‑2 positivity) at admission. All included aneurysms were treated endovascularly according to institutional protocols. Patients were monitored in a neurointensive care unit for a minimum of 12–14 days to ensure early detection of complications such as delayed cerebral ischemia. Radiological grading: The extent of subarachnoid blood on the initial CT scan was assessed using the modified Fisher score (mFS). To ensure consistency, each scan was independently evaluated by two experienced neuroradiologists, with a third neuroradiologist consulted in case of disagreement. The grading was applied according to the original definition [6]: Grade 0: No SAH or intraventricular hemorrhage (IVH), Grade 1: Minimal SAH with no IVH, Grade 2: Minimal SAH with IVH present, Grade 3: Thick SAH with no IVH, Grade 4: Thick SAH with IVH (focal or diffuse).

Patients were monitored in neurointensive care units for at least 12–14 days to enable early detection of complications, including delayed cerebral ischemia (DCI). All patients received oral nimodipine (60 mg six times daily) from day one for vasospasm prevention. Daily transcranial Doppler (TCD) monitoring was performed. In cases of suspected macrovascular vasospasm or delayed cerebral ischemia (DCI), indicated by TCD results or clinical deterioration [30], MR imaging and angiography were conducted. If MR results were inconclusive, DSA was performed, and intra-arterial nimodipine was administered if needed.

Demographic and clinical data collected included age, sex, risk factors (hypertension, diabetes, smoking), blood pressure and laboratory values on admission, baseline World Federation of Neurological Surgeons (WFNS) score and modified Fisher scores [6], and interventions (mechanical ventilation, external ventricular or lumbar drainage). It is known that the modified Fisher Scale (mFS) demonstrates only moderate interrater reliability among neurointensivists/neuroradiologists, largely due to inconsistent understanding and application of its criteria [30]. The mFisher score ranges from 0 (no blood) to 4 (focal or diffuse thick SAH with intraventricular hemorrhage [6]. To avoid inconsistent results, all admission CT scans used for calculating the modified Fisher Score (mFS) were analyzed by two independent, trained neuroradiologists. In cases of disagreement, a third neuroradiologist—blinded to the clinical data and measurements—was consulted for evaluation. Functional outcomes were assessed using the modified Rankin Scale (mRS), with a favorable outcome defined as mRS 0–3 and an unfavorable outcome as mRS 4–6. The 3-month mRS was obtained via structured phone interviews or during follow-up visits. DCI was diagnosed according to established criteria [31] as clinical deterioration or new ischemic lesions on imaging within 6 weeks of admission without other identifiable causes. Although this broad time frame is recommended for research definitions, in clinical practice DCI most commonly occurs within 3–14 days after aSAH [6, 9]. MVS was diagnosed by narrowing of cerebral vessels visualized on MR or DSA and confirmed by an independent neuroradiologist.

Systemic or central nervous system infections were defined by the presence of clinical symptoms (e.g., fever > 38 °C), elevated inflammatory markers (C-reactive protein, procalcitonin > 0.5 ng/mL), and positive diagnostic tests (e.g., chest X-ray, blood/CSF/urine cultures). The control group (*n* = 50) consisted of 30 healthy individuals and 20 patients with unruptured, untreated intracranial aneurysms. No significant differences in baseline serum CLDN3 or CLDN5 levels were observed between these two subgroups (*p* > 0.05). Therefore, they were combined into a single control cohort for analyses. These individuals underwent serum sampling and screening for exclusion criteria to ensure comparability. Laboratory testing was performed to rule out ongoing infections that could confound biomarker analysis.

### Serum collection and detection of CLDN3 and CLDN5 by ELISA

The serum required for the CLDN-3 and CLDN-5 measurements was collected on the 1 st (D1), 5th (D5), and 9th (D9) days following the aSAH, from an arterial sampling cannula. The samples were centrifuged at 4000 rpm for 10 min, then immediately placed in a −80 °C freezer until analysis. Serum levels of CLDN3 and CLDN5 were measured using commercially available sandwich ELISA kits (Enzyme Linked Immunosorbent Assay, EH1342 and EH2839; Wuhan Fine Biotech Co., Ltd., China). Serum samples were diluted 1:2 in the sample diluent provided in the kit. Each assay was performed according to the manufacturer’s instructions. Briefly, 100 µl of standards and samples were added to wells pre-coated with a specific monoclonal antibody and incubated at 37 °C for 90 min. Following a washing step to remove unbound material, 100 µl of biotin-labeled detection antibody was added and incubated at 37 °C for 60 min. After further washing, streptavidin-HRP conjugate was added and incubated for 30 min at 37 °C. Color development was achieved using a TMB substrate, and the reaction was stopped with the STOP solution of the kit. Absorbance was measured at 450 nm using a microplate reader. The concentration of CLDN3 and CLDN5 was calculated from a standard curve generated using standards.

### Statistical analysis

The data were analyzed using the SPSS 25.0 (SPSS Statistics v22.0; IBM Corp., Armonk, NY, USA). Graphs were drawn using the GraphPad Prism 10.0 (GraphPad Software, Inc., Boston, MA, USA). Descriptive statistics were utilized to summarize baseline characteristics and clinical variables. Continuous variables were assessed for normality and presented as mean ± standard deviation (SD) if normally distributed (e.g., age), or as median with interquartile range (IQR) for non-normally distributed data (e.g., WFNS score, mFisher score, laboratory parameters such as C-reactive protein, creatinine, white blood cell count, and neutrophil-to-lymphocyte ratio). Categorical variables (e.g., sex, hypertension, diabetes, smoking status, aneurysm location, and clinical interventions) were expressed as absolute frequencies and percentages. Group comparisons involved appropriate parametric or non-parametric tests based on variable distribution (Mann-Whitney U tests for continuous variables, and Chi-square or Fisher’s exact tests for categorical variables). The Kruskal–Wallis test was utilized for comparing quantitative data across multiple groups.

## Results

### Patients characteristics

The study included 200 patients with an average age of 57.8 years (± 12). The study flowchart detailing patient inclusion and exclusion criteria has been provided as a separate supplementary material file, Suppl. T1. A significant proportion of patients were female (71%), with over half suffering from hypertension (52.6%) and 30.6% being smokers. The median WFNS score, was 2 (IQR 1–4), while the median mFisher score was 3 (IQR 2–3). Aneurysms were most commonly located in the anterior communicating artery (33.3%) and middle cerebral artery (28.3%). The median C-reactive protein level was 16 mg/L (IQR 4–55), while the median creatinine level was 60 µmol/L (IQR 49–71), indicating relatively stable kidney function. Half of the patients (51.5%) required a lumbar drain, and 45.9% needed mechanical ventilation. Delayed cerebral ischemia occurred in 20.1% of cases, and 34.5% of patients developed infections during their treatment. Based on the modified Fisher score, the number of patients was as follows: mFS1 *n* = 39, mFS2 *n* = 50, mFS3 *n* = 67, mFS4 *n* = 44. There was no significant difference in age between the control group (*n* = 50) and the patient population (ctrl: 54 ± 14 ys vs. patients:57.8 ± 12 ys, *p* = 0.112), and the gender distribution (ctrl: female 68% vs. patients 71%, *p* = 0.463) also did not differ between the two groups. The detailed characteristics of study population can be found in Table 1.

**Table 1:** Patients characteristics

Variable	Total (*n* = 200)
Age (mean ± SD)	57.8 ± 12
Female, N (%)	142 (71)
Hypertension, N (%)	105 (52.6)
Diabetes, N (%)	21 (10.7)
Smoking, N (%)	61 (30.6)
WFNS, median (IQR)	2 (1–4)
mFisher score, median (IQR)	3 (2–3)
Aneurysm location, N (%)	
internal carotid artery	18 (8.9)
middle cerebral artery	57 (28.3)
anterior communicating artery	67 (33.3)
posterior communicating artery	22 (11.1)
anterior cerebral artery	7 (3.9)
vertebrobasilar	29 (14.4)
C-reactive protein^a^, mg/L, median (IQR)	16 (4–55)
Creatinine^a^, µmol/L, median (IQR)	60 (49–71)
White blood cell count^a^, G/L, median (IQR)	10.7 (9–13)
Neutrophile-lymphocyte ratio^a^, median (IQR)	5.5 (4–10)
Lumbal drain, N (%)	103 (51.5)
Mechanical ventillation, N (%)	92 (45.9)
Decompressive craniotomy, N (%)	20 (10.2)
Extraventricular drainage, N (%)	30 (45.2)
Delayed cerebral ischemia, N (%)	40 (20.1)
Infection, N (%)	69 (34.5)

*N* number, *SD* standard deviation, *WFNS* world federation of neurological surgeons score, *IQR* interquartile range

### Level of CLDN3 and CLDN5 in patients and controls

**Fig. 1:**
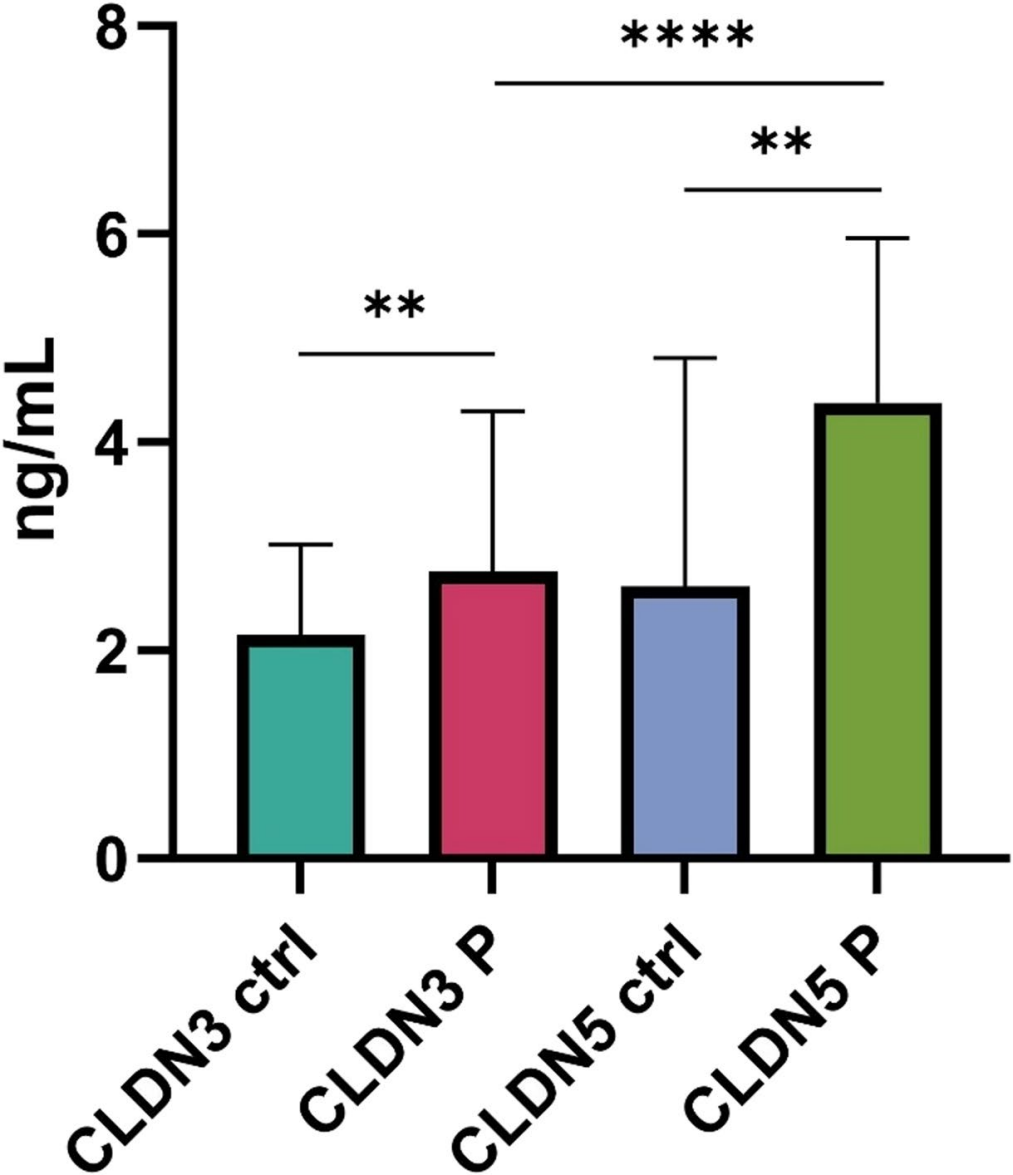
Serum CLDN3 and CLDN5 levels in controls (*n* = 50) and in patients with aSAH (*n* = 200). Patient values represent the overall median and interquartile range (IQR) across day 1, day 5, and day 9 measurements, as no significant differences were found between these time points. CLDN3 = claudin-3; CLDN5 = claudin-5; ctrl = control; P = patients. ****p < 0.0001; **p < 0.01. Data are shown as median with interquartile range. Mann–Whitney U test was used for comparisons. *p* < 0.01, *p* < 0.0001

Serum levels of both CLDN3 and CLDN5 were significantly elevated in patients compared to control subjects. CLDN3 levels showed a modest but statistically significant increase (*p* < 0.01), while CLDN5 levels in patients were markedly higher than in controls (*p* < 0.0001). Additionally, patient CLDN5 levels were significantly higher than patient CLDN3 levels (*p* < 0.01), Fig. 1.

### Serum CLDN3 and CLDN5 on days 1, 5, and 9 post-aSAH

**Fig. 2:**
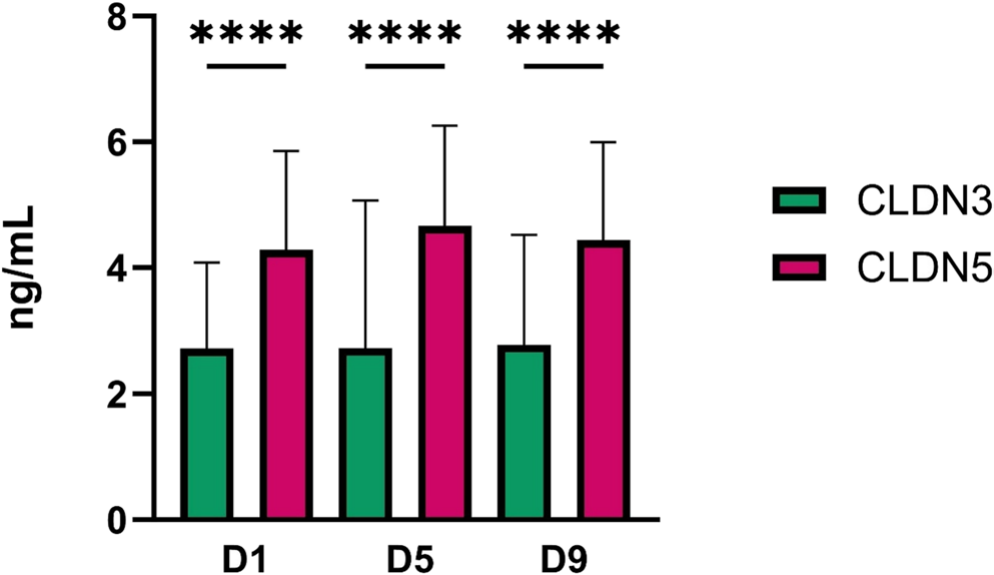
Serum levels of CLDN3 and CLDN5 on days D1, D5, and D9 following aSAH. CLDN3, claudin-3, CLDN5, claudin-5, D1, Day 1, D5, Day 5, D9, Day 9, aSAH, aneurysmal subarachnoid hemorrhage, **** *p* < 0.0001. Data are shown as median with interquartile range. Differences between time points were analyzed using the Kruskal–Wallis test with post hoc pairwise comparisons

Serum concentrations of CLDN3 and CLDN5 were measured on days 1 (D1), 5 (D5), and 9 (D9) following aneurysmal subarachnoid hemorrhage (aSAH), Fig. 2. At each time point, CLDN5 levels were significantly higher than CLDN3 levels (*p* < 0.0001), as indicated by the bar graph and statistical annotations. CLDN3 levels remained relatively stable across all three time points, with means slightly below 3 ng/mL. In contrast, CLDN5 levels consistently exceeded 4 ng/mL on D1, D5, and D9, showing a marked elevation compared to CLDN3. When analyzed separately, neither CLDN3 nor CLDN5 showed significant variation across day 1, day 5, and day 9 (*p* > 0.05 for all timepoint comparisons), indicating stable levels over time. Control CLDN3 and CLDN5 levels are shown in Fig. 1. Figure 2 focuses on the longitudinal pattern within aSAH patients only; control samples were not included in this timecourse analysis because control subjects did not undergo serial sampling.

### Association between the modified Fisher score and serum CLDN3 and CLDN5 levels

**Fig. 3:**
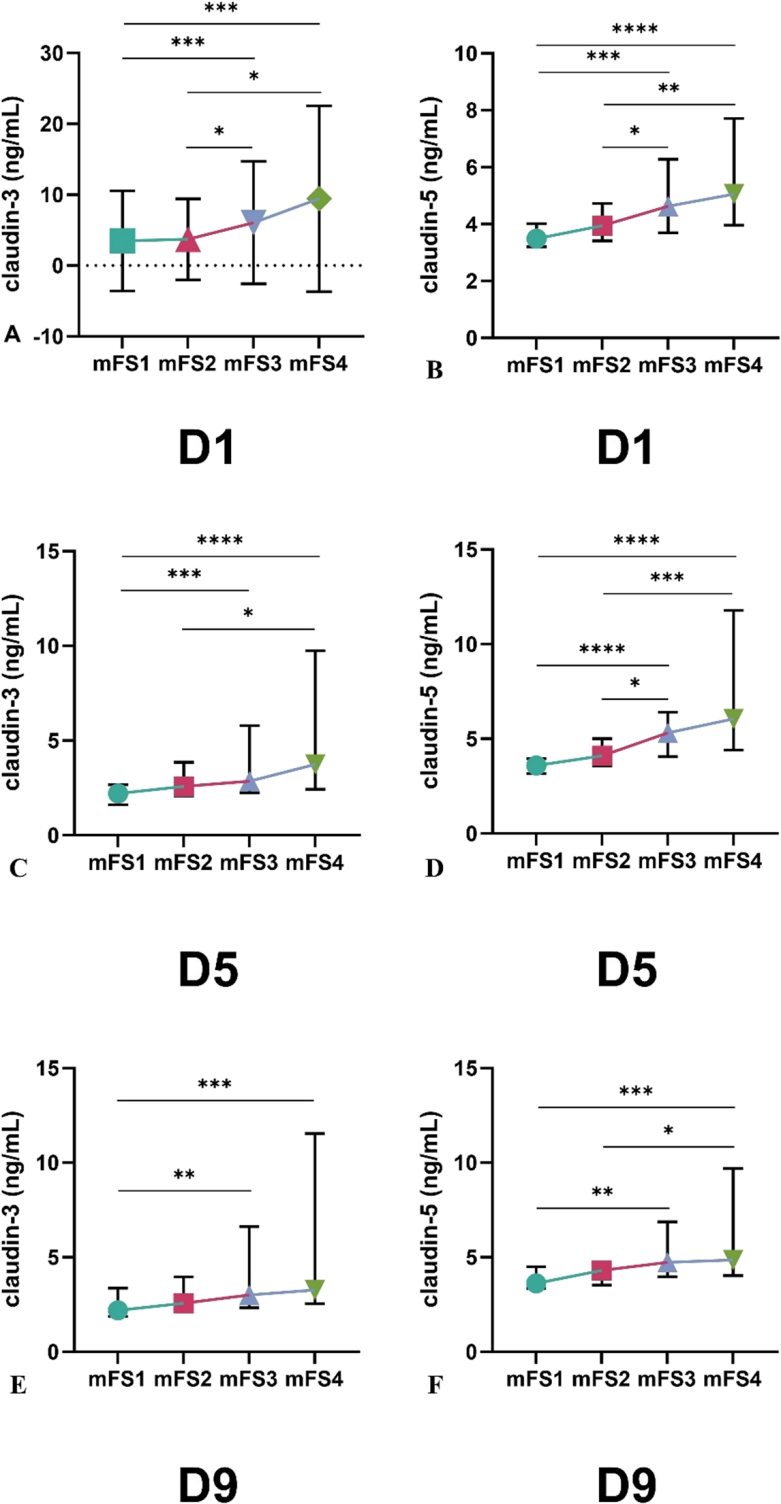
Serum CLDN3 and CLDN5 values on days D1, D5, and D9 after aSAH based on the modified Fisher score. **A** CLDN3 on day D1, **B** CLDN5 on day D1, **C** CLDN3 on day D5, **D** CLDN5 on day D5, **E** CLDN3 on day D9, and **F** CLDN5 on day D9. CLDN3, claudin-3, CLDN5, claudin-5, D1, Day 1, D5, Day 5, D9, Day 9, mFS, modified Fisher score, number of patients in each group: mFS1 *n* = 39, mFS2 *n* = 50, mFS3 *n* = 67, mFS4 *n* = 44. Data are presented as median with interquartile range. Kruskal–Wallis test followed by Bonferronicorrected post hoc comparisons was used

Serum levels of CLDN3 and CLDN5 were analyzed on days 1 (D1), 5 (D5), and 9 (D9) after aneurysmal subarachnoid hemorrhage (aSAH) in relation to patient outcomes based on the modified Rankin score (mFS), Fig. 3. On Day 1 post-aSAH, CLDN3 levels (A) were significantly higher in patients with mFS3 and mFS4 compared to those with mFS1 and mFS2 (*p* < 0.05, *p* < 0.001, respectively), with the highest values observed in the mFS3 group. Similarly, CLDN5 levels (B) increased progressively with worsening mFS scores, showing significant differences across all comparisons. On Day 5, CLDN3 levels (C) were significantly elevated in mFS3 and mFS4 groups compared to mFS1 and mFS2, with the most robust differences between mFS1 vs. mFS3 (*p* < 0.001) and mFS1 vs. mFS4 (*p* < 0.0001). CLDN5 levels (D) also followed a clear trend of increase with worse outcomes, with mFS4 showing the highest levels and significant differences observed between mFS1 vs. mFS2 (*p* < 0.05), mFS1 vs. mFS3 (*p* < 0.001), and mFS1 vs. mFS4 (*p* < 0.0001). By Day 9, CLDN3 levels (E) remained significantly elevated in patients with higher mFS scores, particularly between mFS1 and mFS3 (*p* < 0.01) and mFS1 and mFS4 (*p* < 0.001). CLDN5 levels (F) on D9 followed a similar pattern, with significant differences between mFS1 vs. mFS2 (*p* < 0.01), mFS1 vs. mFS3 (*p* < 0.05), and mFS1 vs. mFS4 (*p* < 0.001).

The serum CLDN3 and CLDN5 levels, in contrast to the mFS, do not show any association with the WFNS score or the 3-month mRS scale values. Similarly, no correlation was observed with demographic variables (age, sex), medical history (diabetes, hypertension, smoking), admission laboratory parameters (CRP, WBC, NLR), or complications (DCI).

The data can be seen in Table S1.

## Discussion

In our current study, we found the following key results: (i) Serum CLDN3 and CLDN5 levels were significantly higher in the aSAH group compared to the control group, (ii) total CLDN5 levels were significantly higher than serum CLDN3 levels at all three measurement time points (D1, D5, D9), (iii) neither total CLDN3 nor total CLDN5 levels showed significant differences across the individual time points, and finally, (iv) both molecules showed a significant positive correlation with the modified Fisher score in aSAH patients.

A study by Kazmierski et al. [29] found that higher serum levels of CLDN5, as well as increased CLDN5/Zonulin1 ratios, were significantly associated with clinical deterioration caused by hemorrhagic transformation. It is supports our findings by demonstrating that serum tight junction proteins, including CLDN5, are reliable indicators of blood-brain barrier (BBB) disruption and are predictive of hemorrhagic complications in ischemic stroke. Both studies reinforce the role of CLDN5 as a clinically relevant biomarker of BBB integrity. Jiao et al. [32] supports the findings of our current research by demonstrating that CLDN5 plays a critical role in maintaining blood-brain barrier (BBB) integrity and is significantly disrupted following cerebral ischemia. Their evidence of tight junction protein downregulation in response to vascular injury aligns with the elevated serum CLDN5 levels observed in aSAH patients in our study, indicating BBB compromise. Jäkel et al. [33] found decreased microvascular CLDN5 levels in cerebral amyloid angiopathy-associated ICH underscores the sensitivity of CLDN5 to vascular injury. This aligns with the elevated serum CLDN5 levels seen in aSAH patients in the our study, highlighting CLDN5 as a consistent marker of BBB disruption regardless of hemorrhage subtype. In our study, CLDN3 and CLDN5 levels showed a strong correlation with the modified Fisher Score (mFS), which indicates the extent of hemorrhage, but not with the WFNS score, which reflects clinical severity. A recently published study [34] similarly found that CLDN5 levels may indicate the degree of blood-brain barrier disruption, however, CLDN5 concentrations did not correlate with patients’ neurological or functional status in the acute phase, suggesting its primary utility lies in reflecting structural BBB damage rather than clinical condition. Matrix metalloproteinases (MMPs), play a key role in the pathophysiology of subarachnoid hemorrhage by contributing to blood-brain barrier disruption and early brain injury [35–37]. A study by Yang et al. [38] showed that activation of MMPs opens the BBB by degrading tight junction proteins (claudin-5 and occludin) and increases BBB permeability after stroke, and this may be one of the potential mechanisms in aSAH patients for the proportional increase in CLDN3 and CLDN5 levels with the extent of the hemorrhage. The temporal pattern of CLDN5 elevation on Days 1, 5, and 9 suggests persistent BBB compromise beyond the acute phase. This mirrors the work of Yang et al. [38], who described prolonged MMP-mediated degradation of CLDN5 in cerebral ischemia, emphasizing its vulnerability in neurovascular injuries​. This study demonstrates significantly elevated serum levels of CLDN3 and CLDN5 in patients following aSAH, with CLDN5 levels consistently exceeding those of CLDN3 at all time points. This finding supports the growing recognition of tight junction proteins as key biomarkers for BBB disruption. Among tight junction components, CLDN5 in particular has been spotlighted in various neurovascular disorders due to its central role in paracellular barrier function. Greene et al. [19] emphasized CLDN5 as the ‘gatekeeper’ of the BBB, and our results reinforce its central role in BBB integrity, showing its consistent elevation in patients with more extensive radiological hemorrhage (higher mFS). Importantly, however, we did not find any association between CLDN5 levels and functional outcomes (mRS) or clinical severity (WFNS score), indicating that elevated CLDN5 reflects structural blood–brain barrier disruption rather than predicting recovery or neurological status. This may reflect increased endothelial permeability and a disrupted neurovascular unit, mechanisms that are well documented in stroke and aSAH pathology.

Interestingly, despite the clear correlation between CLDN3 and CLDN5 with mFS, no association was found with the WFNS score, 3 month mRS, or with common clinical and laboratory parameters (e.g., CRP, WBC, NLR). This suggests that tight junction protein levels may serve as more specific biomarkers of BBB disruption rather than general disease severity or systemic inflammation. LasekBal et al. [34] reported a similar specificity in stroke patients, where CLDN5 and occludin levels varied by stroke type and location but not by neurological status. The disproportionately higher levels of CLDN5 compared to CLDN3 might also reflect differences in their regional expression or molecular stability under inflammatory conditions; indeed, Jiao et al. [32] and Duan et al. [28] emphasized the region and injuryspecific regulation of tight junction proteins, with CLDN5 appearing more responsive to ischemic and hemorrhagic insults, while CLDN3’s role remained less pronounced. This lack of association with WFNS and 3 month mRS may also be explained by the different pathophysiological domains these variables represent. While the mFS reflects the extent of hemorrhage and early BBB disruption, WFNS and mRS integrate a broader spectrum of factors—including secondary injury, systemic complications, and neuroplasticity—that evolve over time and are not captured by a single early biomarker. Blood–brain barrier dysfunction itself is dynamic: advanced imaging studies have demonstrated that BBB permeability abnormalities can predict poor longterm outcomes even in patients without severe early radiographic findings [39], and Chen et al. [40] similarly highlighted early and persistent BBB disruption as a key driver of pathophysiology. Therefore, elevated serum claudin levels primarily reflect the magnitude of early structural BBB injury rather than the complex, timedependent processes that determine clinical severity or longterm recovery.

Although many of our interpretations were supported by studies in ischemic stroke, it is important to note that BBB disruption is also a key feature of hemorrhagic strokes such as intracerebral hemorrhage (ICH). Prior studies, for example Jäkel et al. [33], have demonstrated altered microvascular CLDN5 expression in ICH, underscoring that tightjunction degradation is not unique to ischemia. However, the pathophysiology may differ: in hemorrhagic stroke, direct exposure of brain tissue to extravasated blood and its breakdown products may lead to a more immediate and severe BBB insult compared with the primarily hypoxic–ischemic injury seen in ischemic stroke. Therefore, while our findings align with data from ischemic stroke regarding CLDN3/CLDN5 as markers of BBB dysfunction, they also highlight a pattern of BBB compromise that is consistent with—but potentially more pronounced in—the hemorrhagic setting of aSAH.

Several Limitations of our study should be acknowledged. First, although the study was prospective and involved a relatively large cohort of 200 aSAH patients, it was conducted at a single academic center, which may limit the generalizability of the findings to broader or more diverse populations. Future multicenter studies are needed to validate the utility of CLDN3 and CLDN5 as biomarkers across various clinical settings. Second, while the modified Fisher Score (mFS) was used as a primary radiological correlate of hemorrhage severity, it is known to have only moderate interrater reliability, even among experienced neurointensivists and neuroradiologists. Although our study attempted to mitigate this limitation by involving independent and blinded neuroradiologists, some degree of subjectivity in scoring cannot be fully excluded. Third, the study did not assess cerebrospinal fluid (CSF) levels of CLDN3 and CLDN5, which may more directly reflect central nervous system pathology than serum levels. Future investigations comparing serum and CSF concentrations could help clarify the source and significance of these biomarkers. Fourth, although CLDN3 and CLDN5 levels correlated with the extent of hemorrhage, no association was found with clinical severity scores (WFNS) or functional outcomes (mRS). This suggests that while these proteins may reflect structural blood-brain barrier (BBB) disruption, they may not capture the full complexity of neurological impairment or recovery. Additional biomarkers or multimodal approaches may be needed to assess clinical prognosis more comprehensively. Further limitation of our study is that other markers of neuronal injury (e.g., NSE, S100β, GFAP, HMGB1) were not measured alongside CLDN3 and CLDN5. Future studies integrating these markers may provide complementary information on the relationship between BBB integrity and neuronal injury after aSAH. Lastly, this study did not examine the potential mechanistic pathways leading to tight junction protein release into the circulation, such as matrix metalloproteinase activity or endothelial injury markers. Further mechanistic studies could improve our understanding of the temporal dynamics and regulation of BBB integrity post-aSAH.

## Conclusion

In conclusion, the present results emphasize the potential of CLDN5, and to a lesser extent CLDN3, as dynamic biomarkers for BBB disruption and hemorrhagic injury severity in aSAH. Future studies with longitudinal follow-up and mechanistic insights into tight junction dynamics may elucidate their roles further and pave the way for targeted therapeutic strategies.

## Supplementary Information

Below is the link to the electronic supplementary material.

ESM 1 (DOCX 14.5 KB)

ESM 2 (DOCX 15.5 KB)

## Data Availability

No datasets were generated or analysed during the current study.
