# Supplementary material for: Claudin proteins and hemorrhage severity in aneurysmal subarachnoid hemorrhage: Correlation with modified Fisher score but not functional outcome
Source: Neurosurg Rev. 2025 Oct 20;48(1):725. doi: 10.1007/s10143-025-03829-y (PMC12537756; doi:10.1007/s10143-025-03829-y)
Supplement: Supplementary file 1 — (DOCX 14.5 KB) [file 10143_2025_3829_MOESM1_ESM.docx]

**Study Flowchart: Patient Inclusion Process**

**Total patients assessed for eligibility:** 287

**Excluded (n = 87):**

- Traumatic SAH (n = 16)
- Bleeding due to AVM (n = 7)
- Pregnancy (n = 1)
- Admitted >24 hours after ictus (n = 19)
- No aneurysm treatment (n = 10)
- No signed consent (n = 9)
- Underlying systemic diseases (n = 25)
  - Malignancies (n = 5)
  - Liver/renal insufficiency (n = 6)
  - Chronic lung disease (n = 4)
  - Chronic neurological disease (n = 3)
  - IBD or GI disorders (n = 3)
  - Acute/chronic infection incl. SARS-CoV-2 (n = 4)
  - **Included in final analysis:** 200 patients

All treated endovascularly within 24 hours
