# Supplementary material for: Claudin proteins and hemorrhage severity in aneurysmal subarachnoid hemorrhage: Correlation with modified Fisher score but not functional outcome
Source: Neurosurg Rev. 2025 Oct 20;48(1):725. doi: 10.1007/s10143-025-03829-y (PMC12537756; doi:10.1007/s10143-025-03829-y)
Supplement: Supplementary file 2 — (DOCX 15.5 KB) [file 10143_2025_3829_MOESM2_ESM.docx]

|  | CLDN3 | CLDN5 |
| --- | --- | --- |
| WFNS | 0,086 | -0,005 |
| mRS | 0,086 | 0,003 |
| Age | -0,041 | -0,036 |
| WBC | 0,083 | 0,014 |
| Neutrophile count | 0,087 | 0,042 |
| Lymphocyte count | -0,037 | -0,120 |
| CRP | 0,131 | 0,085 |

Table S2A. Spearman’s correlation coefficients between selected clinical parameters and CLDN3 and CLDN5 expression levels. Values represent correlation coefficients (Spearman’s rho). Positive values indicate a direct correlation, while negative values indicate an inverse correlation. WFNS – World Federation of Neurosurgical Societies grade; mRS – modified Rankin Scale; WBC – white blood cell count; CRP – C‑reactive protein.

|  | CLDN3 | CLDN5 |
| --- | --- | --- |
| Gender | 0.866 | 0.766 |
| Diabetes | 0.228 | 0.587 |
| Hypertension | 0.688 | 0.600 |
| Smoking | 0.328 | 0.651 |
| Delayed cerebral ischemia | 0.875 | 0.699 |

Table S2B. Kruskal–Wallis test p‑values for associations between clinical variables and CLDN3 or CLDN5 expression. Values represent p‑values obtained from Kruskal–Wallis tests comparing CLDN3 and CLDN5 expression across groups defined by each clinical variable. A p‑value < 0.05 was considered statistically significant.
